# Understanding and Exploiting Phage–Host Interactions

**DOI:** 10.3390/v11060567

**Published:** 2019-06-18

**Authors:** Edel Stone, Katrina Campbell, Irene Grant, Olivia McAuliffe

**Affiliations:** 1 Teagasc Food Research Centre, Moorepark, Fermoy, P61 C996 Cork, Ireland; 2 Institute for Global Food Security, School of Biological Sciences, Queen’s University, Belfast, 19 Chlorine Gardens, BT9 5DL Belfast, UK; katrina.campbell@qub.ac.uk (K.C.); i.grant@qub.ac.uk (I.G.)

**Keywords:** bacteriophage, detection, biosensor, food-safety, agriculture, receptor binding protein, endolysin, phage–host interactions

## Abstract

Initially described a century ago by William Twort and Felix d’Herelle, bacteriophages are bacterial viruses found ubiquitously in nature, located wherever their host cells are present. Translated literally, bacteriophage (phage) means ‘bacteria eater’. Phages interact and infect specific bacteria while not affecting other bacteria or cell lines of other organisms. Due to the specificity of these phage–host interactions, the relationship between phages and their host cells has been the topic of much research. The advances in phage biology research have led to the exploitation of these phage–host interactions and the application of phages in the agricultural and food industry. Phages may provide an alternative to the use of antibiotics, as it is well known that the emergence of antibiotic-resistant bacterial infections has become an epidemic in clinical settings. In agriculture, pre-harvest and/or post-harvest application of phages to crops may prevent the colonisation of bacteria that are detrimental to plant or human health. In addition, the abundance of data generated from genome sequencing has allowed the development of phage-derived bacterial detection systems of foodborne pathogens. This review aims to outline the specific interactions between phages and their host and how these interactions may be exploited and applied in the food industry.

## 1. Introduction

Phages are the most abundant microorganisms in the biosphere, with an estimated 4.8 × 10^31^ phage particles present. Phages are present in all areas where bacteria thrive and play a significant role in population dynamics of microbes in the majority of ecosystems and in the evolution of their bacterial and archaeal host cells. As drivers of microbial diversity, phages have sparked interest within the scientific community as means to understand fundamental molecular biology interactions, as vectors of horizontal gene transfer, sources of diagnostic and genetic tools and novel bacterial detection systems [1]. Translated literally, bacteriophage means ‘bacteria eater’. Phages are specific viruses of bacteria that hijack the bacteria’s metabolic mechanisms in order to replicate, which, in the case of lytic phages, subsequently leads to the death of the host cell [2]. Given the distribution and prevalence of phages, it is surprising that they were not identified for close to 40 years following the commencement of significant bacteriological work in Europe and America in the 1880s. The beginning of phage research is usually credited to an unusual observation made by Fredrick W. Twort in 1915. However, the recognition that phages were responsible for Twort’s observation was only made following the pioneering work of Felix d’Herelle [3]. From this discovery evolved many others. Chemical analyses of purified phages revealed that their proteinaceous nature and the presence of phosphorus in these virus preparations suggested a second component, subsequently recognized as nucleic acid. Target theory approaches were employed in order to estimate the size of phages [4]. Experiments were designed to discover the intracellular increase in phage numbers between infection and lysis. It was discovered that phages are obligate intracellular parasites that require a suitable bacterial host cell to multiply and proliferate [5]. Infection begins through phage recognition of and adsorption to a host cell receptor via phage receptor binding proteins (RBP). In the next stage of the process, the viral genome is injected into the host bacterial cell’s periplasm and the host cell’s metabolic pathway is exploited to replicate the viral genome [6]. The formation of new virions then begins and these virions are subsequently released into the surrounding environment [7,8]. Throughout this review, the relationship between the RBPs and receptors of the host cell, and the exploitation of these interactions to create novel diagnostic and preventative techniques will be discussed.

## 2. A Brief Overview of Phage Morphology and Classification

**Figure 1:**
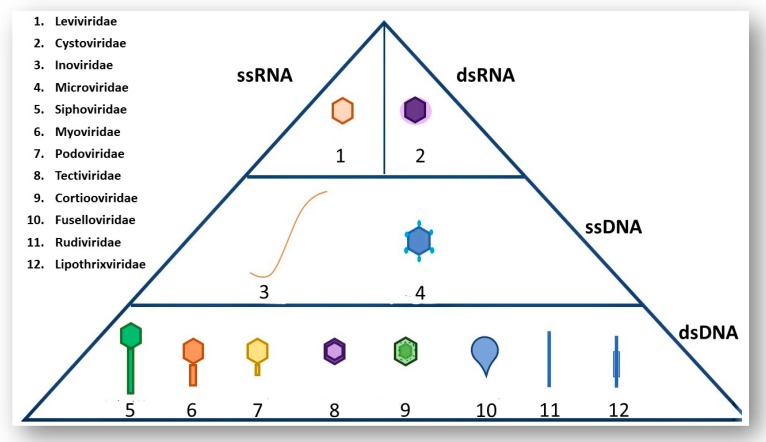
Examples of phage families that are grouped on the basis of their genetic material. The current taxonomy comprises of 22 families of bacterial and archaeal families which can be sourced from the International Committee of Taxonomy of Viruses (ICTV) master list [11].

The majority of known phages belong to the order *Caudovirales* and are tailed phages [9]. The order *Caudovirales* can be further sub-divided into three different families according to tail morphology: *Myoviridae* (long contractile tail), *Siphoviridae* (long non-contractile tail) and *Podoviridae* (short non-contractile tail). The International Committee of Taxonomy of Viruses (ICTV) uses many phage characteristics for classification including phage morphology, physiochemical properties of the virion, nucleic acid content and genomic data [9]. It is important to note the various nucleic acid content that phages may have as this information along with phage morphology are the main determinants of the order and family of a phage; Figure 1 shows examples of different phages and the type of genetic material these phages contain [10].

## 3. The Interaction of a Phage and its Bacterial Host Cell

**Figure 2:**
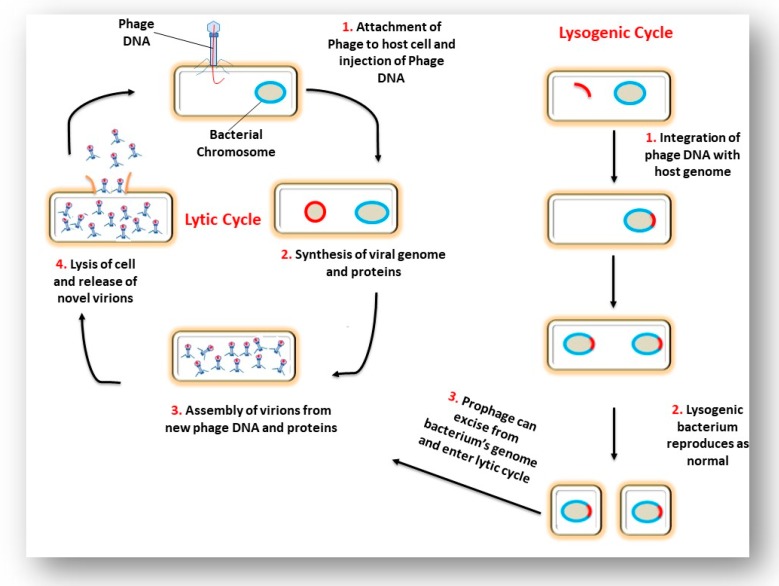
The lytic and lysogenic lifecycles of phages.

Phage life cycles dictate their role in bacterial and archaeal biology [12]. Three life cycles of phages have been reported: lytic, lysogenic and chronic phages. In general, once a virulent phage (a phage that follows the lytic cycle) has attached to its host cell, the phage’s nucleic acid enters the cell and causes the bacterium to produce hundreds of phage copies. This results in the lysis of the cell and the newly formed phages are released into the surrounding environment. Temperate phages (phages that follow the lysogenic cycle) may follow one of two scenarios. The first scenario results in the lysis of the host cell and release of newly formed phages, similar to the lytic life cycle outlined above. In the second scenario, phage DNA may be integrated into the bacterial chromosome. The integrated DNA (prophage) is non-infectious and replicates as part of the bacterial chromosome. Incorporation of the phage DNA into the bacterial chromosome can be beneficial for the evolution of the bacteria as useful genes may be transferred to the bacteria [12]. These prophage-mediated changes have been termed lysogenic conversion [13]. In this state of symbiosis, both phage and the host cell experience an increased level of fitness. Under UV light or certain chemical treatments, the prophage is excised and causes the bacteria to produce phage particles. Figure 2 depicts the lytic and lysogenic lifecycle. The third life cycle is the chronic lifecycle which occurs in archaeal viruses and some filamentous and temperate phages. These viruses do not cause cell disruption or cell death, but instead the newly formed virions are continuously released from the cell. The infected host cells are capable of growing but at a much slower rate [14].

**Figure 3:**
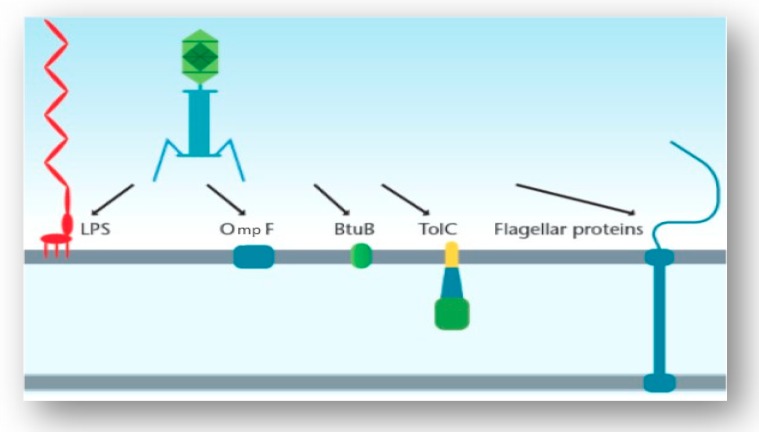
Examples of the possible host cell receptors a tailed phage generally may adsorb to on bacteria.

Generally, the infection process begins with the phage attaching to the surface of the host cell via particular host cell surface receptors. As a consequence of infection, the genetic material of the phage is injected into the cytoplasm of the bacterial cell. The initiation of phage infection is triggered by the specific recognition between the phage’s RBPs located at the tip of the tail and a receptor located on the surface of the host cell. This specificity is directly related to the specificity of adsorption, which correlates to the structure of receptors located on the host’s cell surface [15]. The localization, volume and density of these receptors play a pivotal role in the recognition process. Cell surface receptors recognized by the phage may include protein receptors (OmpA and OmpC), lipopolysaccharide (LPS) receptors, receptors located in capsular polysaccharides (Vi-antigen), pili and flagella (Figure 3) [16]. Proteins that act as receptors for phages may carry out a variety of functions in the host cells (i.e., enzymes, transport proteins, structural proteins, porins and flagella) [16]. Once successful binding to the host receptor has occurred, a conformational alteration in the phage’s baseplate occurs and consequently results in sheath contraction and injection of the phage’s nucleic acid into the host cell.

To begin the replication process, a phage may first have to overcome a variety of carbohydrate boundaries present on the surface of the bacterial cell. These carbohydrate moieties include capsular polysaccharides which can mask the host cell receptors [17,18] and extracellular polysaccharides that may be secreted during biofilm production [18]. Phages have evolved a variety of carbohydrate active enzymes (polysaccharide depolymerases, a common component of the tail in bacteriophages [19]) that function to recognize, bind and degrade carbohydrate components and gain access to a once inaccessible host cell receptor. The binding of phages to these host cell receptors is discussed in detail in Section 3.3 and Section 3.4. In accordance to their mechanism of action, phage depolymerases can either be hydrolases or lyases, each of which causes the breakdown of polysaccharides into soluble oligosaccharides. The vast majority of hydrolases are members of the O-glycosyl hydrolases group which function by using a water molecule to cleave the O-glycosidic bonds of the polysaccharide. To form soluble oligosaccharides, lyases cleave a glycosidic bond through β-elimination resulting in the introduction of a new double bond and, unlike hydrolases, they do not use water [18]. Hydrolases include sialidases that breakdown capsular polysialic acid and rhamnosidases that hydrolase O-antigen of LPS. Lyases include pectin lyases which degrade extracellular polysaccharides and hyaluronidases that degrade hyaluronate-based capsules [7].

If following a lytic life-cycle, the newly formed phages within the bacterial cell must lyse the cell in order to release these virions into the surrounding environment. Tailed phages accomplish this lysis through the use of the phage-encoded enzyme endolysin and the protein holin [20]. Endolysins are peptidoglycan (PG) degrading enzymes synthesized during the late phase of gene expression in the lytic cycle. At a time where it is critical for lysis to occur, endolysins degrade the bacterial cell wall from within [21]. The most commonly found catalytic domains in these enzymes have muramidase or amidase activity. For phages infecting Gram-negative bacteria, the endolysin is generally a monomeric and globular polypeptide. Endolysins of Gram-positive phages are usually modular in structure with the catalytic domain (N-terminal) connected to the cell binding domain (CBD) (C-terminal). Research carried out by Loessner and team [21] on the phages A188 and A500 that infect the Gram-positive bacterium *Listeria monocytogenes (L. monocytogenes)* indicated that the CBDs of these phages function in directing the phage endolysins Ply118 and Ply500 to their substrates present on the bacterial cell wall. Endolysins are granted access to the bacterial PG through holins which oligomerize in the cytoplasmic membrane and thus create small pores in the membrane and allow the endolysins to reach their substrates [22]. Degradation of the outer membrane of the bacterial host is usually required for lysis also. This is carried out by a spanin complex which is composed of an outer membrane lipoprotein (o-spanin) and an integral cytoplasmic membrane protein (i-spanin) [23].

### 3.1. Endolysins from Phages Infecting Gram-Negative Bacteria

Holins produced by the T4 phage must provide a pathway for endolysins to degrade the cell wall causing lysis of the bacterial cell and release of the newly formed virions into the surrounding environment. Holins can be classified into two groups: canonical holins and pinholins. Canonical holins form large holes allowing the release of pre-folded, non-specific endolysin from the cytoplasm into the periplasmic space. Pinholins create much smaller holes that function only to depolarize the cell membrane [24]. Holins from the T4 phage are canonical holins (T holin) that allow the release of endolysin from the cytoplasm. The T4 phage endolysin is known to have lysozyme activity that degrades host peptidoglycans. Once the canonical holins produce holes in the bacterial wall, the endolysin is free to begin the degradation of the host cell wall [25].

Research regarding the lysis of *Eshcerichia coli* by the T4 phage shows that lysis can be indefinitely delayed when in the lysis inhibition state (LIN). LIN occurs when a T4-infected cell becomes superinfected five minutes or more after the original infection. Infection begins as normal with irreversible infection occurring and a subsequent penetration of the outer membrane by the central tail tube. Yet, in the LIN state, the cytoplasmic membrane is not punctured, and virions are ectopically ejected into the periplasm of *E. coli*. This leads to the activation of a small protein called RI (antiholin) which then inhibits the T holin of the T4 phage and thus, inhibits the lysis of the host cell [26].

Virion-mediated “lysis from without” (LO_V_), as opposed to lysis caused by exogenously applied lysin (lysin-mediated LO (LO_L_)), can be defined as a phage’s potential to lyse bacteria without first infecting. LO_V_ is a phenomenon observed in T4, as well as other T-even phages, occurring as a consequence of phage penetration through the bacterial cell envelope during adsorption [27]. In T4 phages, phage penetration is directed by gene product (gp)5, encoding a tail-associated lysozyme [28]. When the numbers of phages adsorbed to individual bacterial cells is low, then the damage caused by gp5 is relatively slight and does not lead to premature bacterial lysis. However, when higher phage numbers adsorb, the cell wall damage caused can result in lysis at “weak points” in the bacterial wall [29]. A lack of phage progeny as a result of this lysis phenomenon can have consequences for phage biocontrol/therapy.

The study of the T4 phage endolysin and the lysis process paved way for research regarding other phages and their endolysins. Rodríguez-Rubio [30] reported the characterization of the *Salmonella* phage endolysin gp110. Conserved domain analysis revealed that the protein is modular in structure and contains an N-terminal PG_binding_1 domain (pfam01471) and a C-terminal DUF3380 domain (pfam11860). The PG_binding_1 domain has repeated motifs (DGIFGKAT and DGIAGPKT); Rodríguez-Rubio [30] states that this characteristic is usual for proteins that interact with repetitive structures such as peptidoglycan. The DUF3380 domain is located in bacteria that are commonly associated with the PG_binding_1 domain and is a member of a family of uncharacterized proteins. Through biochemical analysis of DUF3380 using PG the team show that the domain has *N*-acetylmuramidase activity and cleaves the β-(1,4) bonds between *N*-acetylmuramic acid and *N*-acetylglucosamine in the sugar backbone of PG. This domain also shows low homology with lysozyme [30].

### 3.2. Endolysins from Phages Infecting Gram-Positive Bacteria

As a result of the antibiotic resistance epidemic, much interest has been generated in relation to using phage endolysins as novel antimicrobials. Although there is much interest around these enzymes, very little is known regarding their interaction with the bacterial cell wall. Eugster and team sought to have a better understanding around these interactions using the *Listeria* phage endolysin PlyP35 with the carbohydrates present in teichoic acid polymers on the peptidoglycan [22]. The CBD of the *L.monocytogenes* endolysin PlyP35 recognizes the N-acetylglucosamine (GlcNAc) residue at position C4 of the RboP subunit [22]. The team highlights that the binding of the CBD of this endolysin could be prevented through the removal of wall teichoic acids (WTA) polymers from the cell wall. Through a genetic investigation, it was determined that the *L. monocytogenes* genes lmo2549 and lmo2550 function in the decoration of WTAs with GlcNAc. If either gene is inactivated, resulting in no production of GlcNAc, there is no binding to the CBD PlyP35 [22]. Similarly, the interaction of endolysin Lyb5 from *Lactobacillus fermentum* phage ΦPYB5 and the surface of lactic acid bacteria was investigated by Hu and team [31]. Sequence analysis of endolysin Lyb5 indicated that the C-terminus of this protein (Ly5C) is involved in bacterial cell wall binding due to the presence of three putative lysin motif (LysM) repeat regions. The N or C terminus of LysC was fused with GFP_uv_ and the resulting fusion proteins were expressed in *E. coli.* Following incubation, GFP_uv_ was successfully displayed on the surfaces of *Lactococcus lactis*, *Lactobacillus casei*, *Lactobacillus brevis*, *Lactobacillus plantarum*, *Lactobacillus fermentum*, *Lactobacillus delbrueckii*, *Lactobacillus helveticus* and *Streptococcus thermophilus* cells. When mixed with chemically pretreated *Lactococcus lactis* and *Lactobacillus casei,* an increase in fluorescent intensity was observed in comparison to non-pretreated cells, which suggests that peptidoglycan is the binding ligand for Ly5C. This set of experiments indicates that Ly5C may be a novel anchor for construction of a surface display system for lactic acid bacteria [31].

### 3.3. Interaction of the T4 Phage and its Bacterial Host Cell (Receptors for Attachment)

**Figure 4:**
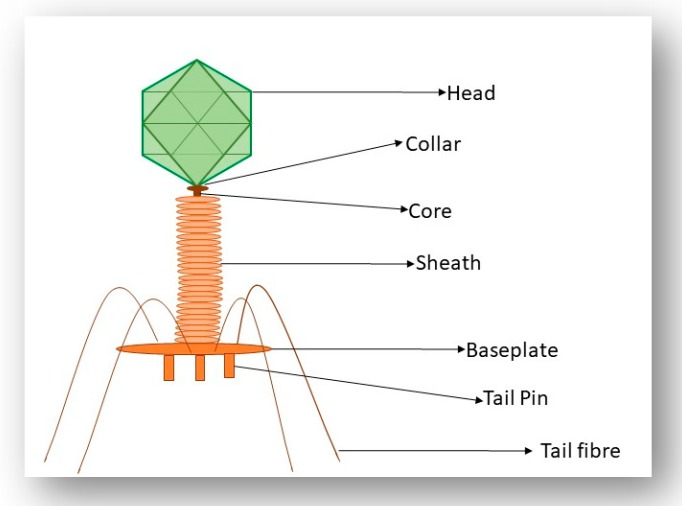
The structural components of a tailed phage depicting the location of the main components of the T4 phage.

The recognition and infection process that is employed by the *Escherichia coli (E. coli)* phage T4 has been used as a model for the study of phage–host interactions in Gram-negative bacteria [32]. T4 is a member of the *Myoviridae* family of the *Caudovirales* order and has an exclusively lytic lifecycle. Recognition of the host cell occurs through the long tail fibers (LTFs) which are attached to the phage tail (Figure 4). The T4 LTFs are a complex trimer of gp34, a monomer of gp35 (proximal segment), a trimer of gp36 and a trimer of gp37 (distal segment). The C-terminal domain of gp37 is responsible for the recognition of the host bacteria. For efficient functioning of the LTFs, additional chaperone proteins are required. Gp38 functions as a chaperone protein in the T4 phage and is necessary for the trimerization of gp37 [33]. The LTFs can bind to both a LPS receptor on the host cell or the protein receptor, outer membrane porin C (OmpC), located on the surface of the *E. coli* cell [34]. In the outer membrane of *E. coli,* OmpC exists as a trimer. In a monomer of OmpC, there is a pore that is formed of 16 beta-barrels and eight loops that connect each β-sheet [34]. Significant research has focused on the binding of the T4 phage to LPS and OmpC. Results from these experiments indicate that the T4 phage adsorbs to *E. coli* via two different modes, OmpC-dependent and OmpC-independent [34]. A study performed by Washizaki et al. [35] shows that the distal tail of phage T4 does not seem to bind to LPS in the presence of OmpC, but instead binds to OmpC directly. The preliminary results of this study support the hypothesis that there is direct binding between OmpC and the distal tail, where the amino acid substitution at a phenylalanine located in the extracellular loop 4 of OmpC causes the inability of T4 adsorption [35]. This result allowed the authors to hypothesize that the binding of the distal tail to OmpC needs simultaneous interaction between respective monomers of OmpC and the distal tail. By conducting these mutational studies, the authors suggest that the top surface of the LTFs of T4 interacts with LPS and the lateral surface interacts with OmpC [35]. Prehm et al. also suggested that the glucose region of the LPS molecule may not be required for the expression of the receptor function and OmpC may not be required at all once the glucose region is exposed at the distal end of the LPS. It may be said that OmpC and the glucose residue of LPS can replace each other in the interaction with gp37 adhesion in the T4 phage [36].

Following LTF binding to these host cell receptors, a recognition signal is sent to the baseplate which results in the extension of the short tail fibers, which irreversibly bind to the receptors. When unbound to a host cell, the LTFs of T4 are folded upwards against the tail, neck and head domains. However, under optimal conditions for infection, an LTF can be released from the neck and head and scavenge the surroundings for a suitable host receptor site. Once a suitable binding site is located, adsorption of phage to a host cell occurs, until a second, and up to six, LTFs bind to the host cell. Once three or more LTFs successfully bind, the chosen host cell is highly likely to be a suitable replication host and the LTFs irreversibly bind to the host cell. Subsequently, the base plate alters its conformation from hexagonal to a star-shaped conformation and the short tail fibers extend, releasing their carboxy-terminal ends to bind tightly to the core region of LPS. The outer tail sheath contracts, driving the inner tail tube through the bacterial outer membrane and periplasm, allowing the end of the inner tube to interact with the bacterial inner membrane [37]. Phage DNA is now ejected into the bacterial periplasm, primed to direct synthesis of new phage particles [34].

### 3.4. Interactions of Other Phages and Their Gram-Negative Host Bacterial Cell (Receptors for Attachment)

Like the T4 phage, the broad host range *Salmonella* phage S16 also binds to the LPS and OmpC of its host cell using its LTFs [33]. The S16 phage is a member of the *Caudovirales* and belongs to the family *Myoviridae*. The composition of the S16 LTF includes five proteins gp34–gp38 extending from the baseplate to the tail fiber tip. The distal segment of the S16 LTF is likely to be structurally similar to the structure reported for the T2 phage, in which the gp38 acts as the protein adhesin which caps a trimeric gp37 β-helix. Through the use of purified S16 LTFs and appropriate null mutants, Marti et al. [33] assessed the binding of S16 green fluorescent protein (GFP)–LTF fusion proteins to the mutant cells. The wild-type revealed an even decoration of the bacterial surface by the fluorescent LTF. Removal of OmpC (ϪompC) totally abolished the decoration by S16 GFP–LTF, demonstrating that OmpC is necessary for sufficient recognition and binding of S16 LTF to *Salmonella* host cells. Furthermore, the ability of the whole S16 phage to bind to *Salmonella* Typhimurium (*S.* Typhimurium) DT7155 ϪompC resulted in a reduction of binding compared to the wild-type (47% vs. 98%). The results from Marti et al. [33] indicate that the primary receptor of phage S16 is OmpC. While knowing the vital role of OmpC, the possible synergistic relationship between LPS and OmpC cannot be ignored. The mutational analysis carried out by Marti et al. also highlighted the important role of LPS in the binding of S16 LTFs to *Salmonella*. It was shown that deletion of the inner core of LPS attenuates the adsorption of S16 to *Salmonella* (72% vs. 98%). When a double mutant was created where the host cells lacked OmpC and the inner core of LPS, adsorption of phage was further reduced (13%). Interestingly, deletion of OmpC and the LPS outer core allows complete immunity to infection by S16 [33].

For a better understanding of the interaction of the S16 LTFs and its host cell *Salmonella*, Dunne et al. [38] examined the mechanism responsible for the recognition of the host cell by phage S16. The team generated a crystal structure of gp38 and gp37 of phage S16 and determined that gp38 contains a polyglycine type II sandwich, the distal loops of which form putative receptor binding sites. The C-terminal domain of gp38 mediates host specificity and consists of a series of glycine-rich motifs (GRMs) and hypervariable segments (HVSs). These HVSs of gp38 are sensitive to frequent modular reshuffling that result in chimeric adhesins with different host receptor specificity. Again, the decoration of S16 GFP–LTF fusion proteins on different host cells was observed by Dunne and co-authors. They observed variations in cells decorated with GFP–LTF; when OmpC was absent from the cell wall of *S.* Typhimurium, only 10.6% cell decoration by GFP–LTF was observed vs. the wild-type (100%) [38]. Further analysis of the relationship between the gp38 of S16 and OmpC revealed an LTF binding site on the extracellular Loop 5 of OmpC. There is 98% sequence similarity between the OmpC of *Salmonella* Enteritidis (*S.* Enteritidis) and *S.* Typhimurium; however, a three-residue section (227-NAR-229) exists in *S*. Typhimurium which is switched to (227-DEH-229) in *S.* Enteritidis. It is suggested that this three-residue area may be situated in the extracellular loop 5 of OmpC which is responsible for the binding of the S16 to OmpC of the host cell [38].

Structures that are not located in the cell wall of the bacterium have also been proven to act as receptors for certain phages that infect Gram-negative bacteria. These structures include flagella, pili and capsules. In a study carried out by Shin et al. [39], 25 *Salmonella* phages that infect *S.* Typhimurium were isolated and through random mutagenesis experiments, the host cell receptors for these phages were identified. Receptors were identified for all 25 phages and included vitamin B_12_ uptake outer membrane protein (BtuB) (7 phages), LPS-related O-antigen (7 phages) and flagella (11 phages). Through transmission electron microscopy (TEM), the authors showed that phages using LPS-related O-antigen as a receptor were members of the *Podoviridae* family and phages using BtuB and flagella as receptors were members of the *Siphoviridae* family. The mutational analysis provided insight into the specific genes responsible for infection of a specific host cell by the phage. Through deletion of one or both flagellin genes, *fliC* and *fljB*, the phages in this study were categorized into two groups: FI, phages that can only use *fliC* as a receptor, and FII, phages that can use both *fliC* and *fljB* as receptors. It is surprising that just three receptors were identified in this study as many other outer membrane structures are known to be cell receptors in *Salmonella* including FhuA, TolC and OmpC. The complex structure of *Salmonella* LPS may block access of these outer membrane proteins to the phages, and thus these receptors may not be used by these particular phages [39].

A phage that is similar to the phages described by Shin et al. is the phage iEPS5, a *Siphoviridae* which also binds to the flagella of *S.* Typhimurium. [40]. Like Shin et al., [39] Choi et al. conducted a series of mutational analyses to determine the host cell receptor for iEPS5. Of 1700 clones tested, it was found that only five mutant strains were resistant to infection by iEPS5. All five of the resistant strains had insertions in genes involved in flagella biosynthesis. Here, it was found that iEPS5 does not have a preference for either *fliC* or *fljB* but can use either as a receptor [40]. To further investigate the relationship between this phage and the flagellin of *S.* Typhimurium, the group investigated if the motility of the flagella played a role in the adsorption of the phage to this receptor. *motA* and *motB* are integral membrane proteins that make up the stator complex of the flagellar motor in *S.* Typhimurium. The role of the stator complex is as a proton channel and couples proton flow with torque (rotational force) generation [41]. In a Δ*motA* mutant, assembly of a flagellar filament and basal body proceeded but rotation of the flagella did not occur as there is a lack of torque. Using a range of mutants with varying motilities, it was shown that successful infection by iEPS5 is conditional on the degree of motility. Furthermore, it seems that counter-clockwise rotation of the flagella is required for successful binding to this receptor [40].

**Figure 5:**
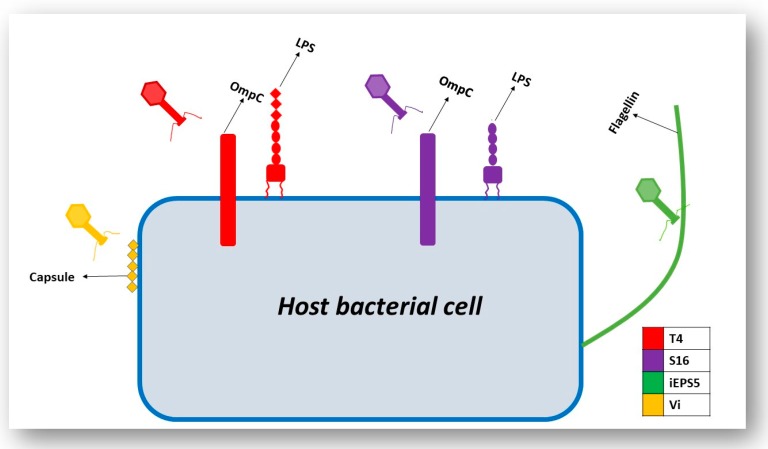
The binding of phages to their host cell receptors. The red phage represents the T4 phage, the phage receptor lipopolysaccharide (LPS) (closest to the phage) and OmpC. The S16 phage is represented in purple, its receptors LPS and OmpC are also in purple. iEPS5 phage and its receptor the flagellin are represented in green and the Vi phage and its receptor sugars of the capsule are depicted in yellow.

Along with LPS, BtuB, flagella and pili, phages that target the Vi capsular antigen of *S.* Typhimurium have also been identified. Pickard et al. aimed to identify the reason that these phages (Vi phages I, III, IV, V, VI and VII) have adapted to use the Vi capsular antigen as a receptor. A conserved protein domain that carries acetyl esterase was found to be associated with a tail fiber gene for all Vi phages [42]. These acetyl groups are located on the Vi exopolysaccharide capsule. A BLASTp analysis identified a putative adhesion region downstream of the acetyl esterase domain in all of the phages. The authors reported that the acetyl esterases of these phages directly target the acetyl modification on the sugars of the capsule itself, which may cause destabilization of the linear Vi fibers due to the loss of hydrogen bond cohesion. By targeting these acetyl groups, efficient infection of *S.* Typhimurium is caused [42]. Figure 5 displays the phages and their host cell receptors discussed in this section.

### 3.5. Phage–Host Interactions in Gram-Positive Bacteria (Receptors for Attachment)

The cell wall of Gram-positive bacteria is a complex structure, composed of various biopolymers including PG, polysaccharides, teichoic acids (glycerol/ribitol-phosphate and amino acids) and (glycol) proteins [15,43]. A significant component of the cell wall of Gram-positive bacteria is PG. It is composed of glycan chains cross-linked through short peptide chains. Secondary polymers, including wall teichoic acids, polysaccharides, or LPXTG-containing proteins, are linked covalently to the PG. Proteins may also be attached non-covalently by recognizing specific motifs of cell wall polymers. Alternatively, they may be organized as a layer outside the cell known as an S-layer. Anchored to the cytoplasmic membrane and inserted in the cell wall are lipoteichoic acids (LTA) which contribute to the functioning of the cell membrane [15]. Phages infecting Gram-positive bacteria generally use a carbohydrate moiety on the surface of the host cell as a receptor, such as cell wall polysaccharides (CWPS) (e.g., *Lactococcus lactis (L. lactis)* phages 936 and p335) and wall teichoic acids (e.g., *L. monocytogenes* phage PlyP35).

#### 3.5.1. *Lactococcus lactis* Phage–Host Interactions

The phages of the Gram-positive lactic acid bacteria (LAB) group have been extensively studied. Members of the LAB group are commonly used in food fermentations where they are used as starter cultures for the production of fermented dairy products including cheese and yogurts. Phages infecting LAB are a real threat in the dairy industry. The main phages infecting *L. lactis* (LAB species commonly used as starter cultures) strains are classified in to three major species: 936, c2 and P335 groups belonging to *Siphoviridae* phage family [44].

Through structural analysis of the RBPs of the phage groups 936 and P335, and genetic analysis of bacteriophage-insensitive mutants (BIMs), there is evidence that phages may use the cell wall polysaccharide (CWPS) of *L. lactis* as a receptor. Phages from species 936 and P335 have proven to have carbohydrate binding properties [45]. Research has shown that the genome of *L. lactis* may contain a single genetic locus which plays a role in CWPS biosynthesis and random insertion mutagenesis resulting in a sedimenting phenotype in liquid medium and insensitivity to infection by particular phages [45]. Subsequently, these authors investigated the CWPS of eight strains of *L. lactis* as a potential receptor for phages in the 936 and P335 groups. Here, an analysis of the genetic locus encompassing the CWPS biosynthesis operon of the eight strains resulted in the identification of a variable region within the strains tested. Located in this variable region were genes encoding glycosyltransferases which display low/no sequence homology within the subgroups (five subgroups of the C-type CWPS, subgroups C_1_–C_5_). The team isolated and purified an acidic polysaccharide from *L. lactis* strain 3107 (C_2_ subgroup) confirming the structural difference between this polysaccharide and the established CWPS of subgroup C_1_ *L. lactis* strain MG1363. Through CWPS swapping experiments and phage challenge assays, it was determined that the CWPS of the subgroup C_2_ is the host cell receptor of two P335 phages, ϕLC3 and TP901-1 [45].

Research regarding the binding of c2 phages to their host cells highlight that a membrane protein is also required for infection of *L. lactis.* Studies have indicated the requirement of a cell membrane protein designated Pip (phage infection protein) as a secondary receptor for c2 phages. Reversible binding occurs for c2 phages when binding to the CWPS and irreversible binding occurs when binding to Pip which leads to the injection of the phages genetic material into the host cell [46]. Phage bLI67 belonging to the c2 family requires a membrane protein for infection of the host cell; the host transmembrane protein YjaE is recognised as a complementary receptor for the bLI67 phage [47].

#### 3.5.2. *Listeria monocytogenes* Phage–Host Interactions

The Gram-positive bacteria *L. monocytogenes* is a foodborne pathogen that is particularly associated with ready-to-eat (RTE) foods that are not cooked or heated before consumption. The burden of listeriosis on the healthcare system and the economic losses and deaths associated with these outbreaks cannot be ignored. Due to the profile of this organism, much interest has now been generated with regards to the interaction of this bacterium and the phages that are specific for this pathogen. Research conducted on the *L. monocytogenes*-specific phages, A118 and P35, has allowed insight into the interaction of the RBPs and teichoic acids located on the cell surface of *L. monocytogenes* [48]. An in-silico analysis was performed to identify potential RBPs from each phage. From this analysis, gp19 and gp20 were identified as putative RBPs for phage A118. Candidate RBPs were also identified for phage P35, which are gp15, gp16 and gp17, due to their location at the end of structural genes. Once these proteins were identified, the team continued to identify the potential receptors that these proteins interact with on the host cell. A GFP label was fused with the potential RBPs of these phages. On the basis of surface marker, such as somatic and flagellar antigens, the genus *Listeria* has 16 serovars and 13 of these are associated among the groups 1/2, 3, 4 and 7 of *L. monocytogenes.* The variation is usually credited to differences in carbohydrate substitution of the polyribitol-phosphate (RboP) subunits of wall teichoic acids (WTA) [22]. Of the serogroups that were tested (1/2a, 1/2b, 1/2c, 4a, 4b, 4c, 5, 6a, 6b and 7) both proteins from A118 and gp16 of P35 were able to fully coat the *L. monocytogenes* serovar 1/2a and 1/2b cells (serotype 1/2a and 1/2b), indicating that these proteins function in host cell recognition [48]. Through a series of mutational analysis, it was determined that rhamnose residues located in the wall teichoic acids are binding ligands for both proteins of A118 phage. The receptor for gp16 in P35 was identified as rhamnose and N-acetylglucosamine [48].

Due to the abundance of both phages and their bacterial host cell it is not feasible to discuss the interaction between every known phage and their corresponding receptors. Table 1 lists common bacterial pathogens and the bacterial receptors for the specific phages mentioned. The aim is to highlight the variety of different host cell moieties that may act as a receptor for phages.

## 4. Exploitation of Phage–Host Interactions

The severe threat to our socio-economic balance and healthcare system from bacterial contamination of food has become a global burden. The World Health Organization (WHO) estimates that 600 million people in the world (approximately 1 in 10 people) fall ill following consumption of contaminated food every year, and of these, 420,000 die. With the changes of food preparation and food styles over recent years, where more processed and RTE foods are available, cooking processes have altered significantly and thus, the risk of consuming food products containing pathogenic bacteria has increased [65]. Foodborne pathogens such as *Campylobacter* spp., *E. coli* O157, *Salmonella* spp. and *L. monocytogenes* are responsible for numerous outbreaks of disease and the recall of food products worldwide. The gold standard for detection of foodborne bacteria is still conventional culture-based diagnostic protocols, due to their sensitivity and the benefit of yielding colonies that can be subjected to further diagnostic tests. However, these methods are time-consuming and labour-intensive [66]. The development of simple to use diagnostics for end product or processing line testing is essential to ensure that the integrity of the food chain is maintained.

Phages have existed alongside their host cells for billions of years and, as described in this review, have evolved systems that may be exploited for our benefit, particularly in the detection of foodborne pathogens. There are a variety of methods available for the detection of bacteria from food, yet the low cost and easy production of large numbers of phages and their high specificity for their target host bacterial cells makes their application for bacterial detection ideal. Recent research has now been focused on the exploitation of phage–host interactions; these exploitations may play a role both in detection of foodborne pathogens and as a biocontrol tool or agent against these bacteria.

Due to the specificity of phages for their target hosts, many applications of phages have been proposed including their potential use to treat acute and chronic infections, as vaccine carriers, and of most relevance to this review, role in food safety and bio-preservation. Below, we discuss how interactions between phages and their host cells may be exploited for detection and elimination of their host cell in the food industry.

### 4.1. Detection of Foodborne Pathogens

Due to the detrimental effects of contamination of food by foodborne pathogens, many systems have been developed, aimed to detect these pathogens. Rapid and reliable detection of foodborne pathogens with high sensitivity is becoming more and more important. The gold standard and most widely used techniques to detect foodborne pathogens on food are conventional culture-based techniques. These methods are reliant on specific media for the enumeration and isolation of viable bacterial cells in food. The benefits of these methods are that they are highly sensitive, cost-effective and may give both qualitative and quantitative information on the number and nature of the bacterial pathogens present. Although these techniques are regarded as the gold standard, it can often take days to successfully identify viable pathogens [67]. Routine procedures for bacterial detection and identification are relatively easy and inexpensive; however, the whole analysis of samples can take up to 72 h, and this length of time for testing is not suited for many cases (i.e., freshly squeezed juices have a shelf life of only 48 h). More rapid methods have been developed with the aim to combat this limitation of time while maintaining a high level of sensitivity and specificity. Such techniques include immunoassays, nucleic acid-based methods and biosensors.

The basis of immunoassays for the detection of foodborne pathogens is antibody–antigen binding. Immunological detection has become more specific, sensitive, reliable and reproducible due to the development of monoclonal antibodies (antibodies with monovalent specificity for one epitope) [68]. A commonly used immunoassay for bacterial detection is the enzyme-linked immunosorbent assay (ELISA). A sandwich ELISA is a practical assay format in which the antigen from a sample is “sandwiched” between two antibodies, the capture antibody on the platform surface and the detection antibody (usually tagged with a fluorescent label). A drawback of these techniques includes the requirement of sample enrichment to obtain detectable levels of pathogens, the requirement of trained personnel for carrying out testing, precise storage conditions of antibodies and the ethical issues associated with the immunization of animals with potentially harmful substances [69]. Another popular technique for detection of bacteria are nucleic acid-based detection techniques, the most popular being polymerase chain reaction (PCR). The principle of PCR is the amplification of target DNA from the pathogen to a level that is detectable, which confirms the presence of DNA from that organism in the sample. Like ELISA-based detection techniques, sample enrichment is usually required for PCR based systems which again adds waiting time for results, this method also requires highly trained personnel. PCR usually cannot differentiate between viable and non-viable cells (unless the sample to be tested is treated with propidium monoazide) [70,71]. Due to the limitation of these techniques, there is a need for novel technology to be rapid, specific, reliable and easy to use. Biosensors present an intelligent alternative to the systems outlined above. A biosensor is a detection system that converts a biological response into a measurable signal. A biosensor consists of four main elements, a bio-recognition element, a signal transduction platform, a signal amplifier and a signal display [69]. The variety of biosensors available and benefits of these systems will be discussed in a later section.

### 4.2. Exploitation of Phage–Host Interactions for the Detection of Foodborne Pathogens

The specific phage–host interactions discussed above may be exploited, particularly for the detection of foodborne pathogens. Due to the issues with more traditional detection methods outlined above, recent research has now been focused on the exploitation of phage–host interactions for the detection of foodborne pathogens [72]. Detection systems based on phage–host interactions do not have the same incapacity as PCR-based systems; as they will detect only living cells these systems can obtain results more rapidly than culture-based techniques and do not require highly trained personnel. The application of whole phages, phage-derived proteins and biosensors for this purpose will now be discussed.

#### 4.2.1. Whole Phages in the Detection of Foodborne Pathogens

The simplest and most direct method to detect foodborne pathogens using whole phages is the generation and enumeration of plaques on a lawn of bacteria in a method known as the phage amplification assay [73]. The sample to be tested is combined with phages that are specific for the pathogen in question, and phages are given time to adsorb and bind to their host cells. If the titer of the phages increases, it correlates to successful binding of the phage to the host cell, leading to lysis and release of progeny phages and thus the presence of the viable target in the food sample if indicated. This type of method was employed by Jung and Ahn [74] for the detection of *Shigella boydii* in artificially inoculated lettuce and chicken breast and in pure culture. The mixtures were treated with 150 µL of FAS at 37 °C for 3 min to destroy any free phages. The assay resulted in the detection of *Shigella boydii* in both single and mixed cultures (*E. coli* O157:H7, *L. monocytogenes* and *Shigella boydii*). No significant difference was noted between single and mixed cultures in the enumeration of plaques which indicates that this assay can specifically identify *Shigella boydii.* However, differences between the number of colonies and plaques were consistently noted in lettuce (6.3 log cfu/mL and 4.9 log pfu/mL) and chicken breast (6.1 log cfu/mL and 6.0 log pfu/mL). The team suggest that this may be due to the adsorption rate of the bacteriophages and the hindrance of the food matrix [74]. Garrido-Maestu [75] used a phage amplification assay (PAA) in combination with qPCR (PAA-qPCR) for identification of *S.* Enteritidis in spiked chicken meat samples. A total of 0.22 fg/µL of pure phage (vB_SenS_PVP-SE2) DNA and 10^3^ pfu/mL of phage particles were detected using the qPCR method. The limit of detection (LOD) of the method was determined to be <10 cfu/25g for 10 h of analysis, including 3 h of pre-enrichment, 6 h of co-incubation, 1 h of DNA enrichment and qPCR analysis. Following the addition of phage to spiked chicken samples, viable plate counts indicated that 8 cfu/25g of *S.* Enteritidis could be detected within 10 h. It was also shown that if the concentration of *S*. Enteritidis is high (10^2^–10^3^ cfu/25g) the detection could be performed following three hours of co-incubation, reducing the detection time to 7 h.

The time required to obtain results is an issue when using phage to detect *Bacillus anthracis* (*B. anthracis)* (12–120 h for clinical identification). Cox and team [76] sought to overcome this issue by using γ phage amplification and lateral flow immunochromatography for the detection of *B. anthracis*. When using LFI (lateral flow immunochromatography) devices to assay phage amplification as a method of bacterial detection it is based on the detection of progeny phages as opposed to the input of phage to initiate infection. The team combined species-specific phage amplification with anti- γ phage antibody-conjugated nanoparticles and reported a bacterial limit of detection (LOD) of 2.5 × 10^4^ cfu/mL for *B. anthracis* (Sterne). Following 2 h a positive result was obtained for an input of B. *anthracis* at 8.0 × 10^5^ cfu/mL. Although this LOD seems quite high the team noted that this is a significant improvement over culture-based detection methods which require 12–120 h to obtain the same result [76].

Another area that focuses on whole-phage detection systems is the use of recombinantly engineered phages (reporter phages). The mechanism of action of these reporter phage systems is based on the modification of phage genomes to incorporate a bioluminescence or fluorescence gene that the phage alone cannot express. Once the phage DNA has been injected into the host cell, bioluminescent/fluorescent proteins are synthesized thereby allowing visual detection of the bacteria. Similarly, reporter phage systems can be created to allow detection based on enzymatic conversion of a chromogenic substrate. The genome of the *E. coli* phage ΦV10 has been exploited for the detection of *E. coli* O157:H7. Modification of the phage to express NanoLuc luciferase (Nluc) allowed bioluminescent-based detection of *E. coli* O157 [77]. This assay detected 5.4 cells in pure culture per assay (in 40 mL) within 7 h when 1.76 × 10^2^ pfu/mL of the reporter phage (ΦV10*nluc)* was employed [77]. When testing for *E. coli* O157 in ground beef enrichment using the NanoLuc phage it resulted in a detection of 4.68 CFU/assay (40 mL) in approximately 9 h [77]. Both results required a pre-enrichment step in sample preparation. This technique was also adapted for the detection of *S.* Typhimurium. The bacterial *luxCDABE* operon was inserted into the *S.* Typhimurium temperate phage SPC32H’s genome. Approximately 20 cfu/mL of *S.* Typhimurium was detected using this bioluminescent reporter phage within 2 h. Results from this experiment also showed that bioluminescent signals increased proportionally to the number of cells present in lettuce, milk and sliced pork, indicating that the reporter phage successfully detects live *S.* Typhimurium [78].

Fluorescent reporter phages usually have integrated fluorescent molecules such as GFP in the genome of the phage so that these phages may be applied to detection assays. The GFP gene is relatively small (approx. 700 bp) and thus, can be easily incorporated into the phage genome [79]. The advantages of using GFP include its stability and autofluorescence which means there is no requirement of a substrate for activation. There are a range of other fluorescent proteins available with a variety of emission/excitation wavelengths which may be chosen for the formation of differently coloured reporter end point or multicoloured end points for multiplex detection of numerous foodborne pathogens [79].

The genome of *Listeria* phage A511 has also been exploited to contain a gene encoding a hyperthermophilic enzyme (β-glycosidase). The *celB* gene encoding the enzyme β-glycosidase was inserted into the phage genome. When *L. monocytogenes* was infected with the reporter phage A511: *celB* it resulted in gene expression and synthesis of a fully functional β-glycosidase enzyme. This particular assay had a detection limit for *L. monocytogenes* of 6 × 10^3^ cfu/mL. This research also showed the practicality of these types of assays, when chocolate milk and salmon were spiked with *L. monocytogenes* the assay detected 10 cfu/g of the bacteria [80]. Although the techniques outlined above can detect foodborne pathogens more rapidly than conventional culture-based techniques (7–9 h), the sensitivity of reporter phage techniques does not compare favourably to culture-based techniques (2 × 10^2^ cfu/mL for reporter phage techniques versus 1 cfu/mL for culture-based techniques).

#### 4.2.2. Phage-Derived Proteins for the Detection of Foodborne Pathogens

Phage proteins that are responsible for the adsorption of the phage to a specific host cell, such as RBPS and CBDs, may also be integrated into systems for the detection of foodborne pathogens. The genome of *Campylobacter jejuni (C. jejuni)* phage NCTC12673 was sequenced and its putative RBP was identified as gp047. This protein was applied to a simple glass slide agglutination assay for the detection of *C. jejuni*. RBPs from this phage showed 100% specificity for *C. jejuni,* 95% for *Campylobacter coli (C. coli)* and 90% for both *C. jejuni* and *C. coli* in pure and mixed cultures. Assays such as this can be performed in minutes and are very cost-effective in comparison to other detection systems available [81]. Phage-derived proteins were also exploited by Denyes [82] and team for the detection of *Salmonella* cells whereby the binding specificity of the LTFs of S16 was harnessed as an affinity molecule. Complexes of recombinant gp37–gp38 LTF were coated onto paramagnetic beads (MBs) for the magnetic separation and enrichment of *Salmonella.* The results obtained showed that 95% of *S.* Typhimurium cells were captured within 45 min from suspensions containing 10–10^5^ cfu/mL. The recovery efficiency of the LTF–MBs was tested on pre-enriched food samples (chicken, infant formula, milk and chocolate milk). The samples were artificially inoculated with 0, 1 to 10, 10, 100 or 1000 cfu/25g or cfu/mL. *Salmonella* was qualitatively detected in all food samples with a limit of 10 cfu/25g or mL. Plating of the bead-captured *Salmonella* resulted in highly sensitive detection of *S.* Typhimurium, however, the technique is not rapid, and the integration of the LTF-based enrichment into a sandwich assay with horseradish-peroxidase (HRP) was investigated to overcome the issue of time. The principle of this assay was based on the HRP–LTF to label the bead-captured *Salmonella,* and the HRP catalyses the conversion of chromogenic 3,3’,5,5’-tetramethylbenzidine substrate leading to the detection of *Salmonella.* It was reported that the colour development in this assay was proportional for *Salmonella* concentrations between 10^2^ and 10^7^ cfu/mL. *S*. Typhimurium cells at a concentration of 10^2^ were detected in 2 h using this assay [82]. Using phage tailed proteins in conjunction with solid phase support (SPS) to simply and rapidly detect foodborne pathogens (*E. coli* O157:H7, *Listeria* spp. and *Salmonella* spp.) in artificially contaminated food samples (ground beef, lentil sprout, soya bean sprout, roast pork, egg and pastry) was investigated by Junillon and team [83]. Here the team functionalized the surface of SPS with specific phage tail proteins to target the pathogen of interest. This SPS is placed into the primary food enrichment bag after stomaching. The sample is incubated for the required time and following this the captured bacteria are detected visually in situ due to the bacterial reduction of the colourless soluble substrate triphenyltetrazolium chloride (TTC) to an insoluble formazan product (intracellular red). When testing foods contaminated with *E. coli* O157:H7 direct observation of the SPS led to a strong positive result (strong reed colour) for lentil sprouts, ground beef and pasteurized and unpasteurized apple juice and a positive result for soya bean sprouts (slightly less red than the other three foods tested) following 22 h of incubation. The SPS was also functionalized using specific *Listeria* spp. phage tail proteins and used to test for the presence of *L. monocytogenes* 4b ATCC 1915 and *L. seeligeri* NSB 22460 in roast pork. Following 40 h of incubation, positive results were obtained for both strains. *Salmonella* Napoli and *S*. Typhimurium were artificially inoculated in egg, pastry and ground beef and gave positive results. When testing *S.* Typhimurium in eggs, a pale red positive result was obtained [83]. The studies above indicate that phage-derived proteins may be applied for the rapid and sensitive detection of foodborne pathogens.

### 4.3. The Use of Biosensors to Detect Bacteria

**Figure 6:**
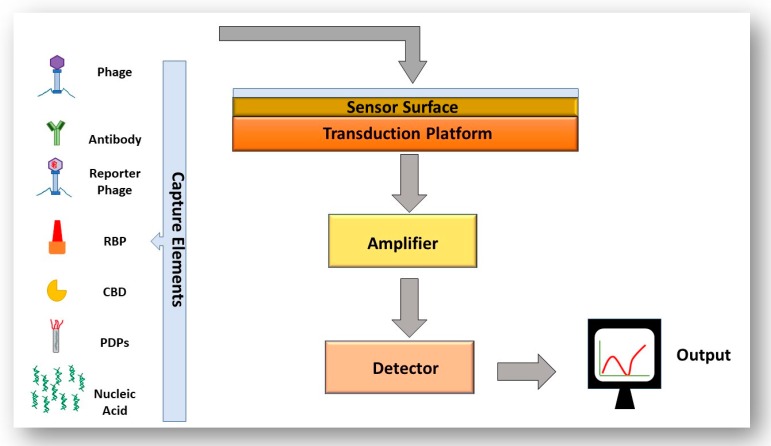
The components of a biosensor, displaying the various recognition elements that may be attached to the sensors surface.

The utilization of phages to act as biorecognition elements in a biosensor is an established idea, to allow rapid, specific and highly sensitive detection of the bacteria in question. Biosensing systems are composed of a recognition element, a sensor surface, a transduction platform, an amplifier, a detector and a signal output. The sensitivity and specificity of the overall system depends on the transduction signal employed and what bio-probe is used. Figure 6 outlines the mandatory components of a biosensor. The majority of systems that utilize phages as recognition elements use the whole phages immobilized onto a solid substrate such as the phage M13 immobilised on a gold surface for detection of *Salmonella* spp., the T4 phage immobilised onto a silver and silicon platform to detect *E. coli* and the BP14 bacteriophage immobilised onto a gold surface for detection of methicillin-resistant *Staphylococcus aureus* (*S. aureus)* [84,85,86].

Along with the use of whole phages, many of the initial sensing systems use surface plasmon resistance (SPR) biosensors based on optical transduction. SPR is a phenomenon that occurs when a beam of polarized light hits a metal surface at the interface of media with a different refractive index. Sensing techniques that use SPR are based on the principle that under specific conditions surface plasmons on the surface of a metal film may be excited by photons and transform a photon into a surface plasmon depending on the refractive index of the adsorbate. The most common geometrical set-up of SPR is the Kretschmann configuration. The incoming polarized light hits the metallic film on the opposite side of where the adsorbate is located. The photons induce an evanescent field into the metallic film. Whenever a plasmon is excited, one photon disappears producing a dip in reflected light at that specific angle. The angle which is dependent on the refractive index of that adsorbate is measured with a charged couple device chip. When the molecule to be detected has absorbed to the surface, the difference between the refractive index of the buffer and refractive index of the molecule can be converted into mass and thickness of the target molecule. In a study carried out by Balasubramanian and team, a biosensor was created to detect *S. aureus* using whole phages and SPR [87]. This sensing system was capable of direct detection of 10^4^ cfu/ml *S. aureus,* without any labelling or amplification steps. In this experiment, the whole phages were immobilized onto the gold surface of a SPREETA sensor via direct physical adsorption, avoiding complex surface chemistry and phage modification. Although this system is a simple to use alternative to label-based systems, SPR based systems have been criticized due to their high LOD (1.3 × 10^7^ cfu/mL) due to the technical limitation of the SPR principle in detecting bacteria (due to their large size). Systems with a lower detection limit would be more applicable to the food testing industry.

Niyomdecha et al. [84] proposed the use of a capacitive flow injection system for the detection of *Salmonella* spp. based on a working electrode modified with a *Salmonella*-specific M13 phage. The mechanism of action of a capacitive measurement is based on the electrical double layer on the surface of a metal electrode. An electrode is fixed with a biosensing element and has a stable capacitance response. Binding of the target bacteria to the biosensing element of the surface results in a decrease in capacitance. This device was capable of detecting 2.5 × 10^2^ to 1.0 × 10^7^ cfu/mL and the LOD was as low as 250 cfu/mL. In order to improve the limit of detection in this system, the team suggested using a lower flow rate or a higher sample volume, both of which would provide a longer contact time between the cell and the analytes. The team tested flow rates between 50–150 µL/min and a sample volume of 250–500 µL/min. A consequence of this is a longer response time. The authors used a lower flow rate of 75 µL/min and a 300 µL sample volume; this resulted in a lower LOD of 200 cfu/mL and a shorter detection time of 40 mins [84]. Although the two systems just described resulted in the accurate detection of foodborne pathogens in a rapid and sensitive manner, the issues associated with using whole phages as the recognition elements cannot be ignored. The incorrect orientation of bacteriophage on the surface of the platform may play a role in the sensitivity of the device. Obtaining the correct orientation of the phages is one major issue that must be overcome by the manufacturers of the device. In addition, it has also been reported that phages lose their activity during drying following fixation on a surface [88]. To overcome the issue of the incorrect orientation of phage on sensor surfaces Anany and team [89] created a novel method for oriented immobilisation of phages based on the differences in charge between the phage’s head (net negative charge) and the tail fibers (net positive charge). The hypothesis here being that the phage heads would attach to a positively charged surface, leaving the tail fibers available to capture the bacteria—*E. coli* O157:H7 and *L. monocytogenes*—in this experiment. The cocktail of bound phage onto a positively charged cellulose membrane was examined to control the growth of *L. monocytogenes* and *E. coli* O157:H7 in RTE foods and raw meats. At 25 °C the *Listeria* phage cocktail which was immobilized onto the cellulose membrane reduced the *L. monocytogenes* count by 1.4 log in 24 h on RTE oven-roasted turkey breast. When carrying out the test at 10 °C under the same conditions it resulted in an undetectable level of *L.monocytogenes* to an undetectable level following 1 day of incubation. The immobilized phage cocktail onto the cellulose membrane however, had no significant effect on the artificially inoculated beef when incubated at 25 °C [89].

Research is now primarily focused on using phage-derived proteins for detection of the host cells. The use of phage RBPs and CBDs are an attractive alternative to the use of whole phages. Since CBDs have a strong affinity and specificity for the target bacteria and can be easily cloned in an *E. coli* expression system, much interest has now been generated in their use in biosensing systems.

Singh et al. [90] conducted research into the detection of *C. jejuni* using phage RBPs as a probe. The group exploited the RBP of phage NCTC 12673 (gp48) for the capture of *C. jejuni* using RBP-functionalized microbeads. The RBPs were placed on a gold surface-based transduction platform, using an SPR based detection system. In this study, the gp48 protein was expressed as a fusion protein with a glutathione S-transferase (GST) tag to aid in its purification. The results also showed that the addition of a GST tag prior to immobilization of gp48 provided optimal orientation on the surface which improves the subsequent host capture in comparison to techniques based on random orientation. RBP functionalized SPR substrates were subsequently used to demonstrate a sensitive and selective detection of *C. jejuni* at concentrations as low as 10^2^ cfu/mL [90]. The binding experiments were performed using pure cultures of *C.jejuni subsp.* strain 11168H which were incubated for 18 h at 37 °C.

## 5. Exploitation of Phages as Biocontrol Agents

For almost a century, phages have been used as antimicrobial agents. In the Western hemisphere, the use of phages for this purpose drastically diminished with the emergence of chemical antibiotics, however, they are still heavily used as therapeutics in parts of Eastern Europe. With the emergence of antibiotic resistance, the identification of novel antimicrobials to combat these resistant strains is more important than ever. The ability of multi-drug resistant (MDR) bacteria to enter the food chain during slaughtering requires efforts to be made for the elimination of these bacteria. MDR bacteria can enter the food chain from the environment via the contamination of ground surface water or through spraying of food crops with water containing MDR bacteria derived from animal and human waste [91]. The use of phages to target these resistant bacterial strains is a promising area of research. Ideally, the candidate phage should have a broad host range and also be exclusively virulent to avoid the risk of transmission of bacterial DNA by transduction. While the use of single phages for detection of a foodborne pathogen is useful due to their specificity, as a biocontrol agent or tool, the use of single phages is ineffective due to their limited host range and the host cell may have systems in place to create resistance to this phage (blocking of phage receptors, production of extracellular matrix, production of competitive inhibitors, preventing phage DNA entry, slicing phage nucleic acids and abortive infection mechanisms) [18]. To negate this issue, a number of options have been investigated including the use of phage cocktails, containing mixtures of phages specific for the target pathogen.

### 5.1. Exploitation of Phages as Biocontrol Agents in Food

The use of phages and their proteins as biocontrol agents or tools may be deemed as a suitable alternative, particularly with the Food Drug Administration (FDA) approval of List-Shield^TM^ (cocktail of *Listeria* phages), the LMP-102 phage preparation for the control of *L. monocytogenes* in RTE foods [92]. Moreover, Listex P100 (phage P100), another anti-listerial agent, was approved by the FDA for the purpose of *L. monocytogenes* control in meats and cheese. Other phage-based food processing aids that have been approved include Salmonelex (cocktail of phages) produced by Micreos Food Safety, and EcoShield (cocktail of phages) and SalmoFresh (cocktail of six phages) from Intralytix. Research was conducted using the broad host range *Salmonella* phage FO1-E2 with the aim to reduce *S.* Typhimurium in RTE foods spiked with this bacterium [93]. These RTE foods were spiked with 1 × 10^3^ *Salmonella* cells and 3 × 10^8^ pfu/g of phage was applied and incubated for 6 days at 8 °C or 15 °C. Following the application of the phage preparation and incubation at 8 °C, no viable *S.* Typhimurium cells were recovered. When incubating the samples at 15 °C, the phages reduced *S.* Typhimurium cell numbers by 3 log cfu/mL in hot dogs and 5 log cfu/ml in turkey deli meat and chocolate milk [93].

#### The Use of Whole Phages and Phage-Derived Proteins as Biocontrol Agents in Foods

Although broad host range phages exist for a variety of bacteria, it can be difficult to isolate broad host range phages for a specific bacterium of interest. Therefore, when phages are investigated as a biocontrol agent in research settings, often it is a combination of phages with narrow and varying host ranges, known as a phage cocktail that is employed. There are many antiviral mechanisms that may be employed by the host cell to evade infection from phages which have resulted in the emergence of phage-insensitive bacteria strains [18]. Phage cocktails containing phages that target different receptors of a host cell may reduce the colonisation of foodborne pathogens in foods without the development of phage-insensitive bacteria [94]. The use of phage cocktails, as opposed to single phages, can moderate and delay the emergence of phage resistance, as demonstrated by Fischer et al. [95]. In this example, the authors compared the application of a single phage and a four-phage cocktail in broilers on reduction of *C.jejuni* and the emergence of bacterial resistance to phages. The percentage of isolates demonstrating resistance to each of the four individual phages ranged from 16.49% to 30.25%. In contract, the percentage of isolates demonstrating resistance to the cocktail was 0.23%, suggesting that the long-term efficacy of phage cocktail application is not seriously compromised by the emergence of resistance.

Bai et al. [96] developed a cocktail of phages to target *S.* Typhimurium in fresh produce using phages that target different receptors of *S.* Typhimurium. Fresh lettuce and cucumber were spiked with *S.* Typhimurium. Twenty-one phages were isolated which recognize five different receptors, the flagella, O-antigen, BtuB, core oligosaccharide region of LPS and OmpC. Treatment with the phage cocktail resulted in a 4.8–5.8 log cfu/cm^2^ viable cell reduction in cucumber and 4.7–5.5 log cfu/cm^2^ reduction in lettuce after incubating for 12 h at room temperature (25 °C) [96]. Research such as this highlights the potential of using phage cocktails as an antimicrobial in RTE food products. Similarly, Coffey et al. examined phages e11/2 and e4/1c as potential biocontrol agents for *E. coli* O157:H7 [97]. The experiment involved the inoculation of sections (20 × 20 cm) of cattle hide with *E. coli* O157:H7 (approximately 10^6^ cfu/cm^2^), which were then treated with a suspension of the phage cocktail. Results showed that following an hour after treatment there was no significant reduction in *E. coli* O157:H7 numbers, however, increased exposure time to the cocktail showed a significant reduction in *E. coli* O157:H7 numbers (1.5 log_10_ cfu/cm^2^ reduction) [97].

The genomes of phages can now be easily sequenced and analysed and the information exploited to identify phage-derived proteins which may themselves be used as biocontrol agents to prevent outbreaks of foodborne illness. Recent years have seen the application of phage-derived proteins in foods with the aim to reduce the growth/kill foodborne pathogens. When applied to lawns of indicator bacteria, the endolysin LysZ5 from *Listeria* phage FWLLm3 could lyse *L. monocytogenes*, *L. innocua* and *L. welshimeri*. With the addition of the protein to soya milk spiked with *L. monocytogenes*, this pathogen concentration reduced by more than 4 log_10_ cfu/mL following a 3 h incubation at 4 °C [98]. Other phage proteins such as a virion-associated peptidase hydrolase (VAPGH)-derived fusion protein CHAPSH3 is obtained from the *S. aureus* phage vB_SauS-phiILA88. CHAPSH3 is a fusion of the VAPGH Hyd5 fused to the SH3 domain from lysostaphin (peptidoglycan hydrolase derived from *Staphylococcus simulans* biovar. *staphylolyticus*). The lytic activity of this protein was tested in milk spiked with 10^4^ cfu/mL of *S. aureus.* Optimal activity was seen at room temperature, with the protein reducing *S. aureus* counts to an undetectable level. Furthermore, results showed that CHAPSH3 is heat stable and retains lytic activity following pasteurization [99].

### 5.2. Exploitation of Phages as Biocontrol Agents in Food Producing Plants

#### 5.2.1. Pre-Harvest Treatment of Food Producing Plants

Pre-harvest and post-harvest treatment of food-producing plants is an area of interest to both the industry and researchers due to the economic loss and the threat of illness caused by foodborne pathogens. Das et al. used a phage cocktail as a pre-harvest prophylactic treatment of Pierce’s Disease (PD), a severe disease of grapevines caused by *Xylella fastidiosa* that infects the xylem which is responsible for transporting water around the plant [100]. In this set of experiments the prophylactic treatment was deemed successful with vines which were not treated with phage displaying symptoms of PD and vines treated with the phage cocktail not displaying any symptoms over the course of the experiment [100]. A similar methodology was applied to defend against *Ralstonia solanacearum* infection (a causative agent of bacterial wilt) in tomatoes, where pre-treatment of tomato seedlings with a cocktail of lytic phages (φRSA1, φRSB1 and φRSL1) reduced penetration, movement and growth of root-inoculated bacterial cells [101].

#### 5.2.2. Post-Harvest Treatment of Food Producing Plants

As previously mentioned Listex P100 a single virulent Listeria phage received approval by the FDA as a food-processing aid and a GRAS (generally regarded as safe) status. The effectiveness of Listex P100 as a post-harvest treatment to control the occurrence of *L. monocytogenes* was investigated by Oliveria et al. [102]. The experiment was carried out using melon, pear and apple products (juices and slices) stored at 10 °C for 8 days. Fruit slices were artificially inoculated with a cocktail of *L. monocytogenes,* using a volume of 15 µL of bacterial suspension at a concentration of 1 × 10^5^ cfu/mL was pipetted onto the well of each fruit wedge. Listex P100 was then pipetted onto the surface of each fruit wedge at a centration of 1 × 10^8^ pfu/mL, 30 mL samples of fruit juices were inoculated with final concentrations of 1 × 10^5^ cfu/mL of *L. monocytogenes* and 1 × 10^8^ pfu/mL of Listex P100 [102]. Treatment was more successful on melon followed by pear; however, no effect on apple products was noted. *L. monocytogenes* counts reduced by 1.50 and 1.00 log cfu/plug for melon and pear slices, respectively. The study discussed above by Bai et al. can also be regarded as a post-harvest treatment of lettuce and cucumber to prevent the growth of *S*. Typhimurium [96].

### 5.3. Exploitation of Phages as Biocontrol Agents in Agricultural Animals

Raw materials used in the food industry which includes both crops and animals are at risk of microbial contamination. Pathogenic bacterial manifestations in agricultural animals can result in a reduction of quality of the food product or a reduction in yield due to the unsuitability of meat from infected animals. As an alternative to antibiotics, much research has been generated to investigate the suitability to treat pathogenic bacterial infections in agricultural animals with phage therapy [73]. Carvalho et al. investigated the efficacy of a phage cocktail to reduce the growth of *C. coli* and *C. jejuni* in chickens by two routes of administration, oral gavage and in feed. When administered orally, the phage cocktail decreased the concentration of both bacteria in faeces by approximately 2 log_10_ cfu/g. This reduction was achieved two days post-phage administration when the phage cocktail was incorporated into the bird’s feed [103]. Similarly, when a phage cocktail was administered to pigs inoculated with *S.* Typhimurium, there was reduction in the titre of *S.* Typhimurium by >1.4 log_10_ cfu/g digesta [104]. Although phage cocktails reduced the concentration of pathogens in these studies, further research is required to determine the correct dosing regimens and the most effective combinations of phages targeting these pathogens [105].

The health benefits of phage lysins were investigated in the treatment of bovine mastitis caused by staphylococci. Fusion proteins (λSA2-E-Lyso-SH3b and λSA2-E-Lysk-SH3b) that contained the streptococcal λSA2 endolysin endopeptidase domain combined with the Staphylococcal cell wall binding domains from either lysostaphin or the endolysin LysK. Both constructs killed 16 different *S. aureus* mastitis isolates, which included penicillin resistant strains. Using 100 µg/mL of both λSA2-E-Lyso-SH3b and λSA2-E-Lysk-SH3b in processed cow milk resulted in a reduction of *S. aureus* by 3 and 1 log units, respectively, within 3 h. Following 1 h however, λSA2-E-Lysk-SH3b permitted the regrowth of *S. aureus*. In a mouse model of mastitis when λSA2-E-Lyso-SH3b or λSA2-E-Lysk-SH3b (25 µg/mL) were applied to the mammary glands *S. aureus* reduced by 0.68 or 0.81 log units. Reduction of *S. aureus* mastitis, gland wet weight and intramammary tumour necrosis factor alpha (TNT-α) concentration was also shown in mouse models [105].

### 5.4. The Pros and Cons of Using Phages as Biocontrol Agents

The use of phages as biocontrol agents in food and agriculture does not stem from a competitive advantage over the list of antimicrobials and bactericides, but rather the absolute necessity for novel agents for the control of pathogenic bacteria in this crisis of antibiotic resistance. Therefore, the treatment of pathogenic bacteria or food spoilage bacteria must not have the same weaknesses as antibiotics, which is why the exploitation of phages as novel biocontrol agents has gained significant interest among researchers and in the industry.

While the narrow host range of many phages may be viewed as a disadvantage by some, this characteristic restricts the number of bacteria where the selection for phage-resistance mechanisms can occur in comparison to the large proportion of bacterial pathogens that can be affected by chemical antibiotics. A variety of narrow host range phages can then be applied as a cocktail with the aim to kill an entire species of a bacterium in comparison to one narrow host range phage killing one bacterium within a species. The narrow host range of most phages can be seen as an advantage as their application has a minimal effect on health protecting natural flora, therefore, the subject will not be prone to superinfections [106]. Another advantage of the application of phages is their composition (mostly nucleic acid), which allows them to be non-toxic. A unique advantage of phages is the occurrence of “auto dosing”, during the infection and killing process phages increase in numbers (dependent on relatively high bacterial numbers) meaning that phages contribute to establishing the phage dose. Other advantages include, single dose potential, low environmental impact and the relatively low cost [106].

Although there are many attractive advantages of using phages as biocontrol agents the disadvantages of their use cannot be ignored. The drawbacks of their use include their narrow and thus, limited host range, the potential transduction of virulent traits from one bacterium to another and the risk for potential development of resistant mutants [107]. Kazi et al. state that another disadvantage is that research focused on the application of phage in food is carried through experiments using artificially inoculated foods which do not entirely reflect the true environments where phages may be applied in industry and agriculture [107]. If phages are to be used as biocontrol agents or as pharmaceuticals the interaction of these protein-based, live biological agents with the animal and human immune systems resulting in a potential harmful immune response cannot be ignored. However, this issue is not unique to phages (i.e., if antibiotics function to lyse bacteria the bacteria may release bacterial toxins in situ and protein-based pharmaceuticals can cause an immune response, drugs that are composed of each of these have previously been approved for use [106]).

## 6. Concluding Remarks

The integrity and stability of our food supply chain is continuously at risk due to the growing global population and the ability of foodborne pathogens to genetically diversify and overcome potentially all known antibiotics. The co-evolution of phages with their bacterial host cell has allowed these microorganisms to recognise and attach to their host cells with extreme specificity. The molecular interaction of phages and their host cells is of great interest to the scientific community as these mechanisms may be exploited for our benefit and for the maintenance of a safe and secure food chain. The molecular and structural composition of foodborne pathogens varies from pathogen to pathogen and thus the interaction of phages with the specific host cell will vary from phage to phage also. Exploitation of these interactions may be applied in areas such as foodborne pathogen detection, alternative antimicrobials and agricultural application. With regard to detection of foodborne pathogens research has investigated genetically modified phages to create reporter assays, incorporation of phage RBPs and CBDs into agglutination assays and incorporation of whole phage or phage proteins into biosensing platforms. Although research around the area of phage-based foodborne pathogen detection systems is promising, there is yet to be the creation of a system either commercially or academically that has the same detection sensitivity as conventional culture-based techniques.

Future research regarding the exploitation of phage–host interactions should focus on the creation of novel systems that match the sensitivity of these culture-based techniques but have the benefit of a fast turnaround time. The use of endolysins as novel antimicrobials is also a promising area of research in the agricultural industry and the food industry to combat pathogenic bacteria that have evolved to acquire genes for antimicrobial resistance. For each of these applications the fundamental understanding of the interaction of the phage and its host cell is vital, if these interactions are to be exploited.

## Figures and Tables

**Table 1:** Examples of phages infecting common bacterial pathogens and their bacterial host cell receptor.

Receptors Localized on the Surface of Gram-Negative Bacteria			
Phage	Host Bacterial Cell	Receptor(s)	Reference
**Sf6**	*Shigella flexneri* (found in contaminated food and water)	OmpA OmpC	[49]
**SfMu**	*Shigella flexneri*	O-antigen of lipopolysaccharide (LPS)	[50]
**KSF-1**	*Vibrio Cholera* (found in contaminated food and water)	Mannose-sensitive hemagglutinin type IV pilus	[51]
**ICP1**	*Vibrio Cholera*	O1 antigen	[52]
**PP01**	*Escherichia coli* O157:H7 (carried by some amphibians, fish and invertebrates)	OmpC	[53]
**ᶲV10**	*Escherichia coli* O157:H7	O157 antigen	[54]
**P22**	*Salmonella* Typhimurium (found in intestinal tract of humans)	O-antigen of LPS	[55]
**9NA**	*Salmonella* Typhimurium	O-antigen of LPS	[56]
**F336**	*Campylobacter jejuni* (found in contaminated food and water)	*O*-methyl phosphoramidate (MeOPN)	[57]
**F341**	*Campylobacter jejuni*	Flagellum	[58]
**JG004**	*Pseudomonas aeruginosa* (found in soil and contaminated water)	O-antigen of LPS	[59]
**Phage K8**	*Pseudomonas aeruginosa*	O-antigen of LPS	[60]
**Receptors Localized on the Surface of Gram-Positive Bacteria**			
**Gamma Phage**	*Bacillusanthracis* (found in soil and often infects livestock)	GamR (LPXTG-harboring protein)	[61]
**AP50c**	*Bacillus* *anthracis*	CsaB	[62]
**ᶲ11**	*Staphylococcus aureus* (found on skin and mucous layers of human and animals)	wall teichoic acids (WTA)	[63]
**ᶲSLT**	*Staphylococcus aureus*	lipoteichoic acids (LTA)	[64]
**A118**	*Listeria monocytogenes*	Rhamnose residues in WTA	[48]
**P35**	*Listeria monocytogenes*	Rhamnose and N-acetylglucosamine	[48]

## References

[1] Clokie M.R., Millard A.D., Letarov A.V., Heaphy S. (2011). Phages in Nature. Bacteriophage.

[2] Hatfull G.F., Hendrix R.W. (2011). Bacteriophages and Their Genomes. Curr. Opin. Virol..

[3] Summers W.C. (2012). The Strange History of Phage Therapy. Bacteriophage.

[4] Khan Mirzaei M., Nilsson A.S. (2015). Isolation of Phages for Phage Therapy: A Comparison of Spot Tests and Efficiency of Plating Analyses for Determination of Host Range and Efficacy. PLoS ONE.

[5] Betts A., Gray C., Zelek M., MacLean R.C., King K.C. (2018). High Parasite Diversity Accelerates Host Adaptation and Diversification. Science.

[6] Miller E., Kutter E., Mosig G., Arisaka F., Kunisawa T., Rüger W. (2003). Bacteriophage T4 genome. Microbiol. Mol. Rev..

[7] Fernandes S., São-José C., Fernandes S., São-José C. (2018). Enzymes and Mechanisms Employed by Tailed Bacteriophages to Breach the Bacterial Cell Barriers. Viruses.

[8] Weitz J.S., Poisot T., Meyer J.R., Flores C.O., Valverde S., Sullivan M.B., Hochberg M.E. (2013). Phage–bacteria Infection Networks. Trends Microbiol..

[9] Ackermann H.-W. (2007). 5500 Phages Examined in the Electron Microscope. Arch. Virol..

[10] Ackermann H.-W. (2009). Phage Classification and Characterization. Methods Mol. Biol..

[11] International Committee for Virus Taxonomy Taxonomic Information. https://talk.ictvonline.org/taxonomy/.

[12] Campbell A. (1988). Phage Evolution and Speciation. The Bacteriophages.

[13] Eichhorn I., Heidemanns K., Ulrich R.G., Schmidt H., Semmler T., Fruth A., Bethe A., Goulding D., Pickard D., Karch H. (2018). Lysogenic Conversion of Atypical Enteropathogenic *Escherichia coli* (aEPEC) from Human, Murine, and Bovine Origin with Bacteriophage Φ3538 Δstx2::cat Proves Their Enterohemorrhagic *E. coli* (EHEC) Progeny. Int. J. Med. Microbiol..

[14] Munson-McGee J., Snyder J., Young M., Munson-McGee J.H., Snyder J.C., Young M.J. (2018). Archaeal Viruses from High-Temperature Environments. Genes.

[15] Bertozzi Silva J., Storms Z., Sauvageau D. (2016). Host Receptors for Bacteriophage Adsorption. FEMS Microbiol. Lett..

[16] Rakhuba D.V., Kolomiets E.I., Dey E.S., Novik G.I. (2010). Bacteriophage Receptors, Mechanisms of Phage Adsorption and Penetration into Host Cel. Pol. J. Microbiol..

[17] Mostowy R.J., Holt K.E. (2018). Diversity-Generating Machines: Genetics of Bacterial Sugar-Coating. Trends Microbiol..

[18] Labrie S.J., Samson J.E., Moineau S. (2010). Bacteriophage Resistance Mechanisms. Nat. Rev. Microbiol..

[19] Yan J., Mao J., Xie J. (2014). Bacteriophage Polysaccharide Depolymerases and Biomedical Applications. BioDrugs.

[20] Latka A., Maciejewska B., Majkowska-Skrobek G., Briers Y., Drulis-Kawa Z. (2017). Bacteriophage-Encoded Virion-Associated Enzymes to Overcome the Carbohydrate Barriers during the Infection Process. Appl. Microb. Biotechnol..

[21] Loessner M.J., Kramer K., Ebel F., Scherer S. (2002). C-Terminal Domains of *Listeria monocytogenes* Bacteriophage Murein Hydrolases Determine Specific Recognition and High-Affinity Binding to Bacterial Cell Wall Carbohydrates. Mol. Microbiol..

[22] Eugster M.R., Haug M.C., Huwiler S.G., Loessner M.J. (2011). The Cell Wall Binding Domain of *Listeria* Bacteriophage Endolysin PlyP35 Recognizes Terminal GlcNAc Residues in Cell Wall Teichoic Acid. Mol. Microbiol..

[23] Berry J., Rajaure M., Pang T., Young R. (2012). The Spanin Complex Is Essential for Lambda Lysis. J. Bacteriol..

[24] Pang T., Savva C.G., Fleming K.G., Struck D.K., Young R. (2009). Structure of the Lethal Phage Pinhole. Proc. Natl. Acad. Sci. USA.

[25] Emrich J., Streisinger G. (1968). The Role of Phage Lysozyme in the Life Cycle of Phage T4. Virology.

[26] Moussa S.H., Kuznetsov V., Tran T.A.T., Sacchettini J.C., Young R. (2012). Protein Determinants of Phage T4 Lysis Inhibition. Protein Sci..

[27] Abedon S.T. (2011). Lysis from Without. Bacteriophage.

[28] Arisaka F., Kanamaru S., Leiman P., Rossmann M.G. (2003). The Tail Lysozyme Complex of Bacteriophage T4. Int. J. Biochem. Cell Biol..

[29] Tarahovsky Y.S., Ivanitsky G.R., Khusainov A.A. (1994). Lysis of *Escherichia coli* Cells Induced by Bacteriophage T4. FEMS Microbiol. Lett..

[30] Rodríguez-Rubio L., Gerstmans H., Thorpe S., Mesnage S., Lavigne R., Briers Y. (2016). DUF3380 Domain from a *Salmonella* Phage Endolysin Shows Potent N-Acetylmuramidase Activity. Appl. Environ. Microbiol..

[31] Hu S., Kong J., Kong W., Guo T., Ji M. (2010). Characterization of a Novel LysM Domain from *Lactobacillus* fermentum Bacteriophage Endolysin and Its Use as an Anchor To Display Heterologous Proteins on the Surfaces of Lactic Acid Bacteria. Appl. Environ. Microbiol..

[32] Mahony J., van Sinderen D. (2015). Gram-Positive Phage-Host Interactions. Front. Microbiol..

[33] Marti R., Zurfluh K., Hagens S., Pianezzi J., Klumpp J., Loessner M.J. (2013). Long Tail Fibres of the Novel Broad-Host-Range T-Even Bacteriophage S16 Specifically Recognize *Salmonella* OmpC. Mol. Microbiol..

[34] Hyman P., van Raaij M. (2018). Bacteriophage T4 Long Tail Fiber Domains. Biophys. Rev..

[35] Washizaki A., Yonesaki T., Otsuka Y. (2016). Characterization of the Interactions between *Escherichia coli* Receptors, LPS and OmpC, and Bacteriophage T4 Long Tail Fibers. Microbiologyopen.

[36] Prehm P., Jann B., Jann K., Schmidt G., Stirm S. (1976). On a bacteriophage T3 and T4 receptor region within the cell wall lipopolysaccharide of *Escherichia coli* B. J. Mol. Biol..

[37] Leiman P.G., Chipman P.R., Kostyuchenko V.A., Mesyanzhinov V.V., Rossmann M.G. (2004). Three-Dimensional Rearrangement of Proteins in the Tail of Bacteriophage T4 on Infection of Its Host. Cell.

[38] Dunne M., Denyes J.M., Arndt H., Loessner M.J., Leiman P.G., Klumpp J. (2018). *Salmonella* Phage S16 Tail Fiber Adhesin Features a Rare Polyglycine Rich Domain for Host Recognition. Structure.

[39] Shin H., Lee J.-H., Kim H., Choi Y., Heu S., Ryu S. (2012). Receptor Diversity and Host Interaction of Bacteriophages Infecting *Salmonella enterica* Serovar Typhimurium. PLoS ONE.

[40] Choi Y., Shin H., Lee J.-H., Ryu S. (2013). Identification and Characterization of a Novel Flagellum-Dependent *Salmonella*-Infecting Bacteriophage, iEPS5. Appl. Environ. Microbiol..

[41] Kojima S., Furukawa Y., Matsunami H., Minamino T., Namba K. (2008). Characterization of the Periplasmic Domain of MotB and Implications for Its Role in the Stator Assembly of the Bacterial Flagellar Motor. J. Bacteriol..

[42] Pickard D., Toribio A.L., Petty N.K., van Tonder A., Yu L., Goulding D., Barrell B., Rance R., Harris D., Wetter M. (2010). A Conserved Acetyl Esterase Domain Targets Diverse Bacteriophages to the Vi Capsular Receptor of *Salmonella enterica* Serovar Typhi. J. Bacteriol..

[43] Malanovic N., Lohner K. (2016). Gram-positive bacterial cell envelopes: The impact on the activity of antimicrobial peptides. Biochima et Biophysia Acta.

[44] InjectionChapot-Chartier M.-P. (2014). Interactions of the Cell-Wall Glycopolymers of Lactic Acid Bacteria with Their Bacteriophages. Front. Microbiol..

[45] Ainsworth S., Sadovskaya I., Vinogradov E., Courtin P., Guerardel Y., Mahony J., Grard T., Cambillau C., Chapot-Chartier M.-P., van Sinderen D. (2014). Differences in Lactococcal Cell Wall Polysaccharide Structure Are Major Determining Factors in Bacteriophage Sensitivity. MBio.

[46] Monteville M.R., Ardestani B., Geller B.L. (1994). Lactococcal Bacteriophages Require a Host Cell Wall Carbohydrate and a Plasma Membrane Protein for Adsorption and Injection of DNA. Appl. Environ. Microbiol..

[47] Millen A.M., Romero D.A. (2016). Genetic Determinants of Lactococcal c2 viruses for Host Infection and Their Role in Phage Evolution. J. Gen. Virol..

[48] Bielmann R., Habann M., Eugster M.R., Lurz R., Calendar R., Klumpp J., Loessner M.J. (2015). Receptor Binding Proteins of *Listeria monocytogenes* Bacteriophages A118 and P35 Recognize Serovar-Specific Teichoic Acids. Virology.

[49] Parent K.N., Erb M.L., Cardone G., Nguyen K., Gilcrease E.B., Porcek N.B., Pogliano J., Baker T.S., Casjens S.R. (2014). OmpA and OmpC Are Critical Host Factors for Bacteriophage Sf6 Entry in *Shigella*. Mol. Microbiol..

[50] Jakhetia R., Verma N.K. (2015). Identification and Molecular Characterisation of a Novel Mu-Like Bacteriophage, SfMu, of *Shigella flexneri*. PLoS ONE.

[51] Faruque S.M., Bin Naser I., Fujihara K., Diraphat P., Chowdhury N., Kamruzzaman M., Qadri F., Yamasaki S., Ghosh A.N., Mekalanos J.J. (2005). Genomic Sequence and Receptor for the *Vibrio cholerae* Phage KSF-1: Evolutionary Divergence among Filamentous Vibriophages Mediating Lateral Gene Transfer. J. Bacteriol..

[52] Seed K.D., Faruque S.M., Mekalanos J.J., Calderwood S.B., Qadri F. (2012). Phase Variable O Antigen Biosynthetic Genes Control Expression of the Major Protective Antigen and Bacteriophage Receptor in Vibrio Cholerae O1. PLoS Pathog..

[53] Morita M., Tanji Y., Mizoguchi K., Akitsu T., Kijima N., Unno H. (2002). Characterization of a Virulent Bacteriophage Specific for *Escherichia coli* O157:H7 and Analysis of Its Cellular Receptor and Two Tail Fiber Genes. FEMS Microbiol. Lett..

[54] Perry L.L., SanMiguel P., Minocha U., Terekhov A.I., Shroyer M.L., Farris L.A., Bright N., Reuhs B.L., Applegate B.M. (2009). Sequence Analysis of *Escherichia coli* O157:H7 Bacteriophage Î¦V10 and Identification of a Phage-Encoded Immunity Protein That Modifies the O157 Antigen. FEMS Microbiol. Lett..

[55] Baxa U., Steinbacher S., Miller S., Weintraub A., Huber R., Seckler R. (1996). Interactions of Phage P22 Tails with Their Cellular Receptor, *Salmonella* O-Antigen Polysaccharide. Biophys. J..

[56] Schmidt A., Rabsch W., Broeker N.K., Barbirz S. (2016). Bacteriophage Tailspike Protein Based Assay to Monitor Phase Variable Glucosylations in *Salmonella* O-Antigens. BMC Microbiol..

[57] Sørensen M.C.H., Van Alphen L.B., Harboe A., Li J., Christensen B.B., Szymanski C.M., Brøndsted L. (2011). Bacteriophage F336 Recognizes the Capsular Phosphoramidate Modification of *Campylobacter jejuni* NCTC11168 #. J. Bacteriol..

[58] Baldvinsson S.B., Sørensen M.C.H., Vegge C.S., Clokie M.R.J., Brøndsted L. (2014). Campylobacter Jejuni Motility Is Required for Infection of the Flagellotropic Bacteriophage F341. Appl. Environ. Microbiol..

[59] Le S., He X., Tan Y., Huang G., Zhang L., Lux R., Shi W., Hu F. (2013). Mapping the Tail Fiber as the Receptor Binding Protein Responsible for Differential Host Specificity of *Pseudomonas aeruginosa* Bacteriophages PaP1 and JG004. PLoS ONE.

[60] Mcshan W.M., Lam J.S., Van Nguyen S., Yang H., Pan X., Cui X., Zhang F., He Y., Li L. (2016). Genetic Evidence for O-Specific Antigen as Receptor of *Pseudomonas aeruginosa* Phage K8 and Its Genomic Analysis. Front. Microbiol..

[61] Gillis A., Mahillon J. (2014). Phages Preying on *Bacillus anthracis, Bacillus cereus, and Bacillus thuringiensis*: Past, Present and Future. Viruses.

[62] Bishop-Lilly K.A., Plaut R.D., Chen P.E., Akmal A., Willner K.M., Butani A., Dorsey S., Mokashi V., Mateczun A.J., Chapman C. (2012). Whole Genome Sequencing of Phage Resistant *Bacillus anthracis* Mutants Reveals an Essential Role for Cell Surface Anchoring Protein CsaB in Phage AP50c Adsorption. Virol. J..

[63] Xia G., Corrigan R.M., Winstel V., Goerke C., Gründling A., Peschel A. (2011). Wall Teichoic Acid-Dependent Adsorption of Staphylococcal Siphovirus and Myovirus. J. Bacteriol..

[64] Kaneko J., Narita-Yamada S., Wakabayashi Y., Kamio Y. (2009). Identification of ORF636 in Phage SLT Carrying Panton-Valentine Leukocidin Genes, Acting as an Adhesion Protein for a Poly(Glycerophosphate) Chain of Lipoteichoic Acid on the Cell Surface of *Staphylococcus aureus*. J. Bacteriol..

[65] Mir S.A., Shah M.A., Mir M.M., Dar B.N., Greiner R., Roohinejad S. (2018). Microbiological Contamination of Ready-to-Eat Vegetable Salads in Developing Countries and Potential Solutions in the Supply Chain to Control Microbial Pathogens. Food Control.

[66] Rohde A., Hammerl J.A., Boone I., Jansen W., Fohler S., Klein G., Dieckmann R., Al Dahouk S. (2017). Overview of Validated Alternative Methods for the Detection of Foodborne Bacterial Pathogens. Trends Food Sci. Technol..

[67] Mandal P.K., Biswas A.K., Choi K., Pal U.K. (2011). Methods for Rapid Detection of Foodborne Pathogens: An Overview. Am. J. Food Technol..

[68] Zhao X., Lin C.-W., Wang J., Oh D.H. (2014). Advances in Rapid Detection Methods for Foodborne Pathogens. J. Microbiol. Biotechnol..

[69] Cunningham A., Campbell K., McAuliffe O. (2018). Bacteriophages and Rapid Detection of Bacterial Pathogens: A Novel Approach. Reference Module in Life Sciences.

[70] Kim S.Y., Ko G. (2012). Using Propidium Monoazide to Distinguish between Viable and Nonviable Bacteria, MS2 and Murine Norovirus. Lett. Appl. Microbiol..

[71] Vesper S., McKinstry C., Hartmann C., Neace M., Yoder S., Vesper A. (2008). Quantifying Fungal Viability in Air and Water Samples Using Quantitative PCR after Treatment with Propidium Monoazide (PMA). J. Microbiol. Methods.

[72] Petty N.K., Evans T.J., Fineran P.C., Salmond G.P.C. (2007). Biotechnological Exploitation of Bacteriophage Research. Trends Biotechnol..

[73] O’Sullivan L., Bolton D., McAuliffe O., Coffey A. (2019). Bacteriophages in Food Applications: From Foe to Friend. Annu. Rev. Food Sci. Technol..

[74] Jung L.-S., Ahn J. (2016). Evaluation of Bacteriophage Amplification Assay for Rapid Detection of *Shigella boydii* in Food Systems. Ann. Microbiol..

[75] Garrido-Maestu A., Fuciños P., Azinheiro S., Carvalho C., Carvalho J., Prado M. (2019). Specific Detection of Viable *Salmonella* Enteritidis by Phage Amplification Combined with qPCR (PAA-qPCR) in Spiked Chicken Meat Samples. Food Control.

[76] Cox R.C., Jensen R.K., Mondesire R.R., Voorhees J.K. (2015). Rapid detection of *Bacillus anthracis* by γ phage amplification and lateral flow immunochromatography. J. Microbiol. Methods.

[77] Zhang D., Coronel-Aguilera C.P., Romero P.L., Perry L., Minocha U., Rosenfield C., Gehring A.G., Paoli G.C., Bhunia A.K., Applegate B. (2016). The Use of a Novel NanoLuc-Based Reporter Phage for the Detection of *Escherichia coli* O157:H7. Sci. Rep..

[78] Kim S., Kim M., Ryu S. (2014). Development of an Engineered Bioluminescent Reporter Phage for the Sensitive Detection of Viable *Salmonella* Typhimurium. Anal. Chem..

[79] Smartt A.E., Xu T., Jegier P., Carswell J.J., Blount S.A., Sayler G.S., Ripp S. (2012). Pathogen Detection Using Engineered Bacteriophages. Anal. Bioanal. Chem..

[80] Hagens S., de Wouters T., Vollenweider P., Loessner M.J. (2011). Reporter Bacteriophage A511::celB Transduces a Hyperthermostable Glycosidase from *Pyrococcus furiosus* for Rapid and Simple Detection of Viable Listeria Cells. Bacteriophage.

[81] Javed M.A., Poshtiban S., Arutyunov D., Evoy S., Szymanski C.M. (2013). Bacteriophage Receptor Binding Protein Based Assays for the Simultaneous Detection of *Campylobacter jejuni* and *Campylobacter coli*. PLoS ONE.

[82] Denyes J.M., Dunne M., Steiner S., Mittelviefhaus M., Weiss A., Schmidt H., Klumpp J., Loessner M.J. (2017). Modified bacteriophage S16 long tail fiber proteins for rapid and specific immobilization and detection of *Salmonella* cells. Appl. Environ. Microbiol..

[83] Junillon T., Vimont A., Mosticone D., Mallen B., Baril F., Rozand C., Flandrois J.P. (2012). Simplified Detection of Food-Borne Pathogens: An in Situ High Affinity Capture and Staining Concept. J. Microbiol. Methods.

[84] Niyomdecha S., Limbut W., Numnuam A., Kanatharana P., Charlermroj R., Karoonuthaisiri N., Thavarungkul P. (2018). Phage-Based Capacitive Biosensor for *Salmonella* Detection. Talanta.

[85] Srivastava S.K., Hamo H.B., Kushmaro A., Marks R.S., Grüner C., Rauschenbach B., Abdulhalim I. (2015). Highly Sensitive and Specific Detection of *E. coli* by a SERS Nanobiosensor Chip Utilizing Metallic Nanosculptured Thin Films. Analyst.

[86] Tawil N., Sacher E., Mandeville R., Meunier M. (2012). Surface Plasmon Resonance Detection of *E. coli* and Methicillin-Resistant *S. aureus* Using Bacteriophages. Biosens. Bioelectron..

[87] Balasubramanian S., Sorokulova I.B., Vodyanoy V.J., Simonian A.L. (2007). Lytic Phage as a Specific and Selective Probe for Detection of *Staphylococcus aureus*—A Surface Plasmon Resonance Spectroscopic Study. Biosens. Bioelectron..

[88] Singh A., Arya S.K., Glass N., Hanifi-Moghaddam P., Naidoo R., Szymanski C.M., Tanha J., Evoy S. (2010). Bacteriophage Tailspike Proteins as Molecular Probes for Sensitive and Selective Bacterial Detection. Biosens. Bioelectron..

[89] Anany H., Chen W., Pelton R., Griffiths M.W. (2011). Biocontrol of Listeria Monocytogenes and *Escherichia coli* O157:H7 in Meat by Using Phages Immobilized on Modified Cellulose Membranes. Appl. Environ. Microbiol..

[90] Singh A., Arutyunov D., McDermott M.T., Szymanski C.M., Evoy S. (2011). Specific Detection of *Campylobacterjejuni* Using the Bacteriophage NCTC 12673 Receptor Binding Protein as a Probe. Analyst.

[91] Walsh C. (2010). The Problem of Antimicrobial Resistance in the Foodchain. https://www.safefood.eu/SafeFood/files/8a/8abb9354-4cc2-49a4-b586-2bf0008eb8cf.pdf.

[92] Mahony J., McAuliffe O., Ross R.P., van Sinderen D. (2011). Bacteriophages as Biocontrol Agents of Food Pathogens. Curr. Opin. Biotechnol..

[93] Guenther S., Herzig O., Fieseler L., Klumpp J., Loessner M.J. (2012). Biocontrol of *Salmonella* Typhimurium in RTE Foods with the Virulent Bacteriophage FO1-E2. Int. J. Food Microbiol..

[94] Tanji Y., Shimada T., Yoichi M., Miyanaga K., Hori K., Unno H. (2004). Toward Rational Control of *Escherichia coli* O157:H7 by a Phage Cocktail. Appl. Microbiol. Biotechnol..

[95] Fischer S., Kittler S., Klein G., Glünder G. (2013). Impact of a Single Phage and a Phage Cocktail Application in Broilers on Reduction of *Campylobacter jejuni* and Development of Resistance. PLoS ONE.

[96] Bai J., Jeon B., Ryu S. (2019). Effective Inhibition of *Salmonella* Typhimurium in Fresh Produce by a Phage Cocktail Targeting Multiple Host Receptors. Food Microbiol..

[97] Coffey B., Rivas L., Duffy G., Coffey A., Ross R.P., McAuliffe O. (2011). Assessment of *Escherichia coli* O157:H7-Specific Bacteriophages e11/2 and e4/1c in Model Broth and Hide Environments. Int. J. Food Microbiol..

[98] Zhang H., Bao H., Billington C., Hudson J.A., Wang R. (2012). Isolation and Lytic Activity of the Listeria Bacteriophage Endolysin LysZ5 against *Listeria monocytogenes* in Soya Milk. Food Microbiol..

[99] Rodríguez-Rubio L., Martínez B., Donovan D.M., García P., Rodríguez A. (2013). Potential of the Virion-Associated Peptidoglycan Hydrolase HydH5 and Its Derivative Fusion Proteins in Milk Biopreservation. PLoS ONE.

[100] Das M., Bhowmick T.S., Ahern S.J., Young R., Gonzalez C.F. (2015). Control of Pierce’s Disease by Phage. PLoS ONE.

[101] Fujiwara A., Fujisawa M., Hamasaki R., Kawasaki T., Fujie M., Yamada T. (2011). Biocontrol of *Ralstonia solanacearum* by Treatment with Lytic Bacteriophages †. Appl. Environ. Microbiol..

[102] Oliveira M., Viñas I., Colàs P., Anguera M., Usall J., Abadias M. (2014). Effectiveness of a Bacteriophage in Reducing Listeria Monocytogenes on Fresh-Cut Fruits and Fruit Juices. Food Microbiol..

[103] Carvalho C.M., Gannon B.W., Halfhide D.E., Santos S.B., Hayes C.M., Roe J.M., Azeredo J. (2010). The in Vivo Efficacy of Two Administration Routes of a Phage Cocktail to Reduce Numbers of *Campylobacter coli* and *Campylobacter jejuni* in Chickens. BMC Microbiol..

[104] Callaway T.R., Edrington T.S., Brabban A., Kutter B., Karriker L., Stahl C., Wagstrom E., Anderson R., Poole T.L., Genovese K. (2011). Evaluation of Phage Treatment as a Strategy to Reduce *Salmonella* Populations in Growing Swine. Foodborne Pathogens and Disease.

[105] Schmelcher M., Powell A.M., Becker S.C., Camp M.J., Donovan D.M. (2012). Chimeric Phage Lysins Act Synergistically with Lysostaphin To Kill Mastitis-Causing Staphylococcus Aureus in Murine Mammary Glands. Appl. Environ. Microbiol..

[106] Loc-Carrillo C., Abedon S.T. Pros and Cons of Phage Therapy. No. 2, 111–114. https://www.tandfonline.com/doi/pdf/10.4161/bact.1.2.14590?needAccess=true.

[107] Kazi M., Annapure U.S. (2016). Bacteriophage Biocontrol of Foodborne Pathogens. J. Food Sci. Technol..

